# Ultrasound-guided midline catheters in the neonatal intensive care unit: a single-center pilot study

**DOI:** 10.1007/s00431-026-07218-5

**Published:** 2026-07-02

**Authors:** Lorenzo Zanetto, Andrea Zamunaro, Daniel Nardo, Antuan Divisic, Fiammetta Della Torre, Sabrina Salvadori, Eugenio Baraldi, Luca Bonadies

**Affiliations:** 1 Department of Women’s and Children’s Health, University of Padova, Via Giustiniani 2, 35128 Padova, Italy; 2 Pediatric Palliative Care - Pain Service, Department of Women’s and Children’s Health, University Hospital of Padova, Via Giustiniani 2, 35128 Padova, Italy; 3 Neonatal Intensive Care Unit, Department of Women’s and Children’s Health, University Hospital of Padova, via Giustiniani, 2, 35128 Padova, Italy

## Abstract

To assess the feasibility and performance of ultrasound-guided midline catheters in term and preterm neonates requiring prolonged intravenous therapy without an indication for central access, and to generate hypotheses on factors influencing catheter outcomes. Single-center prospective observational cohort: 70 consecutive neonates received 2-Fr polyurethane midline catheters in a level IV NICU (March 2024–November 2025). Data were analyzed descriptively; complication-free survival was estimated by Kaplan–Meier, and exploratory subgroup comparisons used Fisher’s exact test. Mean weight at insertion was 2582 g (range 700–4920), including very low-birth-weight infants. Median dwell time was 9 days (range 0–176), corresponding to 1144 catheter-days. The primary composite outcome (complication-free dwell ≥ 7 days or elective removal before day 7) was met in 94.3% of devices. Kaplan–Meier complication-free survival was 98.6% at 3 days and 81.2% at 14 days. Device-related complications occurred in 21.4%, predominantly minor and resolving after removal. Bloodstream infection and thrombosis rates were 2.62 and 0.87 per 1000 catheter-days; no thrombophlebitis occurred. Complication rates did not differ by vein diameter < 2 mm or weight < 1500 g, although the study was underpowered for these comparisons.

*Conclusion*: In this pilot study, ultrasound-guided midline catheters were feasible in neonates, including very low-birth-weight infants and those with small-caliber veins, with predominantly minor complications. These hypothesis-generating findings require confirmation in adequately powered; comparative studies before midlines can be recommended as an alternative to central access or repeated peripheral cannulation.

**Table:** 

**What is Known:** • *Midline catheters may offer an intermediate option between peripheral and central venous access, but neonatal experience, particularly in preterm infants, is limited.* • *Data on the feasibility and performance of midline catheters in the NICU are scarce.*	
**What is New:** • *In this single-center pilot cohort of 70 neonates, ultrasound-guided midline catheters achieved a high rate of complication-free dwell time, with predominantly minor, self-limiting complications.* • *Complication rates did not appear to increase in infants < 1500 g or with veins < 2 mm; these hypothesis-generating observations require confirmation in adequately powered studies.*

**What is Known:**

• *Midline catheters may offer an intermediate option between peripheral and central venous access, but neonatal experience, particularly in preterm infants, is limited.*

• *Data on the feasibility and performance of midline catheters in the NICU are scarce.*

**What is New:**

• *In this single-center pilot cohort of 70 neonates, ultrasound-guided midline catheters achieved a high rate of complication-free dwell time, with predominantly minor, self-limiting complications.*

• *Complication rates did not appear to increase in infants < 1500 g or with veins < 2 mm; these hypothesis-generating observations require confirmation in adequately powered studies.*

## Introduction

Vascular access in the neonatal intensive care unit (NICU) remains a major challenge, balancing effectiveness and expected duration against complications [1]. Neonatal short peripheral cannula (nSPC) are difficult to secure in small, fragile neonates, often require repeated attempts, and are short-lived, so one course of therapy may need several cannulations [2], each adding pain, stress, and infection risk that may impair neurodevelopment in preterm infants [3].

Central venous access devices (CVADs) are essential for prolonged or complex therapies but carry mechanical and infectious risks. The assumption that peripheral devices are inherently safer with respect to bloodstream infection is increasingly questioned: peripheral line–associated bloodstream infection is an under-ascertained entity in neonates whose burden is increasingly recognized [4]. The relative infectious risk of peripheral versus central access is therefore less clear-cut than often assumed.

Long peripheral catheters partly bridge nSPCs and CVADs but, terminating in small peripheral veins, show high infiltration (particularly in neonates < 2 kg) and short dwell time (DT), and cannot carry infusates of moderate-to-high osmolarity or irritant/vesicant drugs [5, 6]. Midline catheters are peripheral devices whose tip terminates proximal to the central veins, classified by tip position rather than by vein depth or caliber [7]; they achieve longer DT than nSPCs, but neonatal use remains very limited, owing to technical challenges and concerns such as the catheter-to-vessel ratio [8, 9]. In adult outpatients, midlines show fewer bloodstream infections than peripherally inserted central catheters [10]; whether this applies to neonates is unproven.

### Objective

To evaluate the feasibility, performance, and complication rates of ultrasound-guided midline catheters in term and preterm neonates undergoing prolonged intravenous therapy (e.g., parenteral nutrition, antibiotics) not requiring a CVAD.

## Methods

This single-center prospective observational cohort included all neonates who underwent ultrasound-guided midline placement in the level IV NICU of Padova between March 2024 and November 2025, irrespective of gestational age or birth weight. For each procedure, we recorded patient characteristics, indication, insertion site, target vein diameter, procedure duration, and dwell time. The study was approved by the local Ethics Committee (Comitato Etico Territoriale Area Centro—Est Veneto: 641n/AO) and performed after parental consent and in accordance with the Declaration of Helsinki.

Devices were classified as midlines by tip position (directed to the axillary or subclavian vein, proximal to the central veins) rather than by the depth of the cannulated vein [7]. NAVIGATE does not yet codify a neonatal midline category, and by length (4–8 cm), our devices overlapped the neonatal long peripheral catheter range, so the designation rests on the more proximal tip [7]. Insertion length was estimated beforehand from external anthropometric measurement; at insertion, position was judged from indirect markers (free blood return and ultrasonographic infusate inflow toward the superior vena cava). Direct tip imaging was not recorded for all patients early in the series; on retrospective review of available imaging, tip position was documentable by ultrasound and/or radiography in 52 of 70 catheters. Given the measured insertion lengths and the short intravascular distances in neonates [9], an axillary/subclavian tip is anatomically plausible for most catheters, though this is an inference; classification cannot be confirmed for a minority, particularly the shortest, and all are reported as midlines with this caveat.

Insertion was performed by a dedicated vascular access team following the NEVAT neonatal vascular access bundle [11]: maximal sterile barrier precautions, skin antisepsis with 2% chlorhexidine in 70% isopropyl alcohol (30-s dry time), a standardized insertion checklist, and cyanoacrylate securement with a semipermeable dressing. Multimodal non-pharmacological analgesia was used [12]; systemic sedation was not routine. Vein selection and venipuncture were ultrasound-guided in real time (15–7 MHz microlinear probe; Philips Affinity 70), with an out-of-plane approach, and a 2-Fr polyurethane catheter (Leaderflex, Vygon; 4, 6, or 8 cm) was then advanced.

The primary outcome was a composite of complication-free DT of at least 7 days or elective removal at therapy completion before day 7. A device-related complication was any non-elective removal attributable to the device (bloodstream infection, thrombosis, extravasation, insertion-site leakage, occlusion, or suspected unconfirmed infection); a precautionary removal for suspected catheter fissure, made without confirmation, was classified as non-device-related. Bloodstream infection was a laboratory-confirmed infection arising > 72 h after insertion with the catheter in situ; catheter origin was not confirmed microbiologically, so neither catheter-related nor central-line–associated (being midlines) criteria were strictly met. Analyses were descriptive (IBM SPSS Statistics 30): continuous variables as median (range), categorical as n (%), bloodstream infection and thrombosis per 1000 catheter-days; complication-free survival was estimated by Kaplan–Meier, censoring elective and precautionary removals. Subgroup comparisons (vein < 2 vs ≥ 2 mm; weight < 1500 vs ≥ 1500 g) used Fisher’s exact test and are exploratory, the study being underpowered.

## Results

**Fig. 1:**
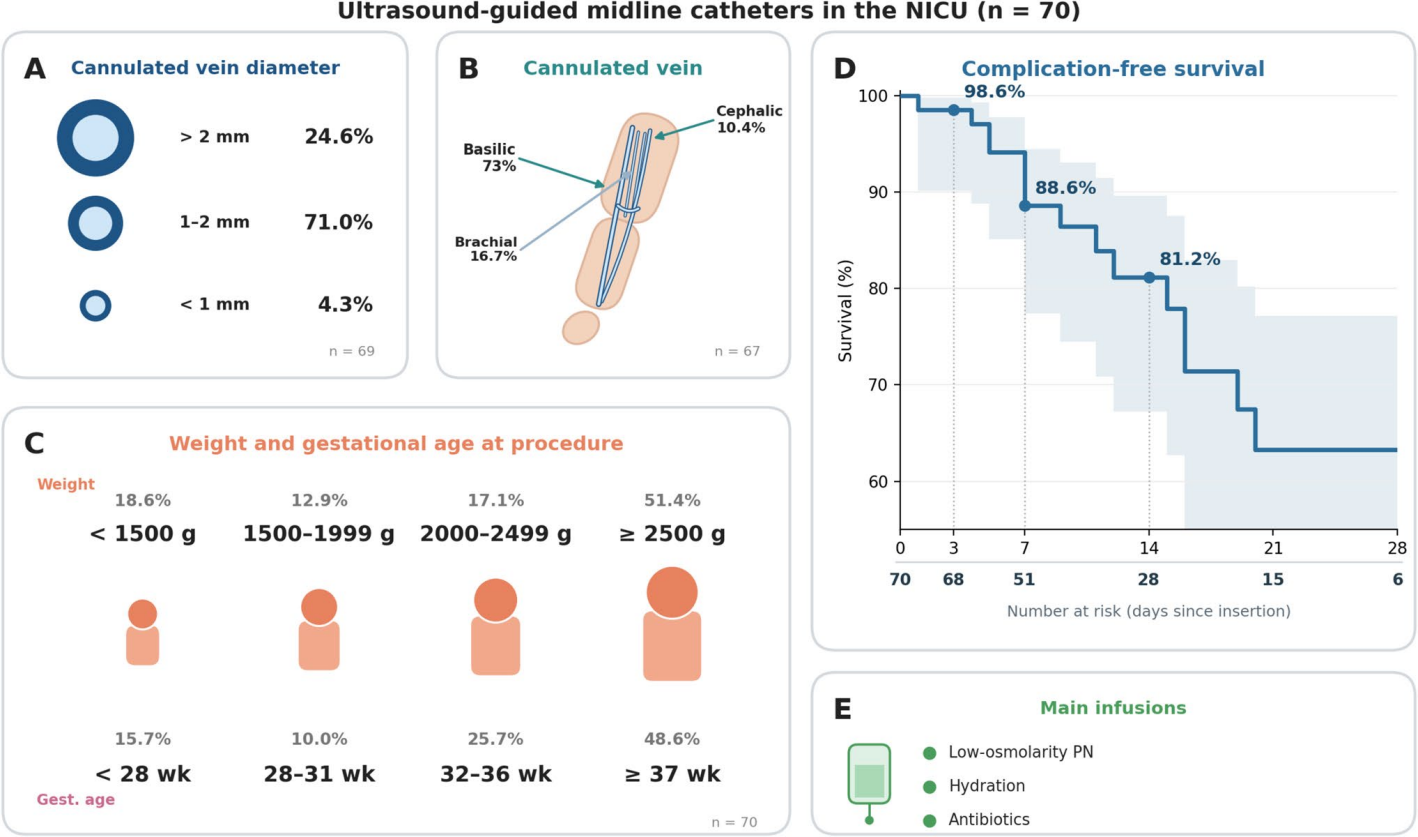
Ultrasound-guided midline catheters placed by a dedicated team in the NICU. **A** Caliber of the cannulated vein; **B** cannulated vein (basilic, brachial, cephalic); **C** gestational age and weight at the time of procedure; **D** Kaplan–Meier complication-free survival at 3, 7, and 14 days; **E** main types of infusion

Seventy consecutive ultrasound-guided midline catheters were analyzed. Mean weight at insertion was 2582 g (range 700–4920) and postnatal age a median of 8 days (mean 25.4; range 0–420); therapy consisted mainly of antibiotics and fluids, including low-osmolarity parenteral nutrition (Fig. 1). The target vein diameter (tourniquet) was available for 69 of 70 procedures (median 1.8 mm, range 0.85–3.0); with the 0.75-mm catheter, the median catheter-to-vessel ratio was 0.42 (range 0.25–0.88), exceeding 0.33 in 58 of 69 cases (84%), and was higher in smaller veins (median 0.54 for veins < 2 mm vs 0.36 for ≥ 2 mm).

The primary composite outcome was achieved in 66 of 70 devices (94.3%). Kaplan–Meier complication-free survival was 98.6% at 3 days, 88.6% at 7 days, and 81.2% at 14 days. Median dwell time was 9 days (range 0–176), corresponding to 1144 catheter-days.

Device-related complications occurred in 15 of 70 catheters (21.4%) and were predominantly minor and resolved after removal: insertion-site leakage (*n* = 3), extravasation (*n* = 3), removal for suspected but unconfirmed infection (*n* = 3), occlusion (*n* = 2), bloodstream infection (*n* = 3), and thrombosis (*n* = 1). Bloodstream infection occurred at 2.62 per 1000 catheter-days and thrombosis at 0.87; no thrombophlebitis was observed. In exploratory analyses, complication rates were similar in infants with veins < 2 mm and ≥ 2 mm (9/41 [22.0%] vs 6/28 [21.4%]; OR 1.03, *p* = 1.0) and in infants < 1500 g and ≥ 1500 g (2/13 [15.4%] vs 13/57 [22.8%]; OR 0.62, *p* = 0.72); given the few events, these comparisons cannot support inference and are hypothesis-generating.

## Discussion

In this single-center pilot study, ultrasound-guided midline placement was feasible across a broad range of neonatal weights and gestational ages, with a high rate of complication-free dwell time and predominantly minor complications. Given the design and sample size, these findings are hypothesis-generating and do not establish device safety.

Current European recommendations frame neonatal peripheral access as a graded choice: short peripheral catheters (n-SPC) and long peripheral catheters (n-LPC) for peripherally compatible therapy of limited duration, and the epicutaneo-caval catheter (ECC) for infusates unsuitable for the peripheral route or for longer courses [1, 11]. Within this framework, peripheral devices are classified by length [7], and a distinct neonatal midline category is not codified. A gap nonetheless persists for the stable neonate who needs peripherally compatible therapy beyond the few days an n-LPC reliably provides, yet has no infusate-based indication for central access. The n-LPC fills this only partially: its more distal tip in small, low-flow veins is associated with high infiltration and short dwell [5]. An ultrasound-guided midline is peripheral by tip position [7] but reaches the higher-flow axillary or subclavian vein, and might extend the peripheral option into this intermediate window: in our cohort, the median dwell of 9 days exceeded the ~ 4 days typically reported for n-LPC [5, 13], plausibly reflecting the more proximal, higher-flow tip. This concerns infiltration and dwell, not infusate tolerance: an ECC remains necessary for hyperosmolar or vesicant infusions, we did not assess infusate compatibility, and our uncontrolled data cannot establish any advantage of midlines over ECCs, which would require direct comparison.

This intermediate-duration role must be weighed against the principle, shared by the DAV-expert algorithm and NEVAT, that device choice be periodically reassessed [1, 11]. Only a small minority of catheters remained in situ for weeks to months (*n* = 4, > 30 days); in these few cases, the indication was reassessed and confirmed both the continued absence of an infusate-based indication for central access (typically low-osmolarity parenteral nutrition) and continued good catheter function. Prolonged dwell warrants re-evaluation rather than being assumed appropriate. There is no direct evidence that a long-dwelling midline is inferior to a central device when the infusate remains peripherally compatible; whether it is an acceptable alternative to a tunneled central catheter in this setting remains a hypothesis for controlled study.

Although 84% of catheters exceeded the conventional 1:3 ratio threshold, thrombosis was rare; this rate, however, was based on clinical detection and may underestimate the true rate, as subclinical events were not sought, and the absence of systematic post-removal surveillance and the limited sample preclude any conclusion that high ratios are safe.

This study has several limitations: it is single-center, observational, and small, and underpowered to detect rare but serious events (symptomatic thrombosis, nerve injury, or delayed bloodstream infection) whose detection requires larger samples and, for thrombosis, systematic ultrasound surveillance that we did not perform. Tip position was not systematically confirmed, leaving residual classification uncertainty, and post-removal vessel patency was not assessed, although successful reinsertion at the same site suggests preserved patency. No randomized trial has compared midlines with nSPCs or ECCs in neonates; future studies should compare procedural burden versus nSPCs and dwell, complications, and infection prevention versus ECCs.

## Conclusions

In this pilot study, ultrasound-guided midline placement was feasible in neonates, including very low–birth-weight infants with small veins, with predominantly minor complications. These findings are hypothesis-generating and do not establish safety; adequately powered comparative studies are needed to define the role of midlines in neonatal vascular access.

## Data Availability

All data are available upon reasonable request to the corresponding author.

## Abbreviations

CVADs: Central venous access devices
DT: Dwell time
ECC: Epicutaneo-caval catheter
NEVAT: Neonatal European Vascular Access Team
NICU: Neonatal intensive care unit
OR: Odds ratio
n-LPC: Neonatal long peripheral cannula
n-SPC: Neonatal short peripheral cannula
